# Evaluation of serum vitamin B12 levels in patients with COVID-19 infection: A case-control study

**DOI:** 10.5937/jomb0-42357

**Published:** 2023-08-25

**Authors:** Yılmaz Sezgin

**Affiliations:** 1 University of Health Sciences Turkey, Istanbul Training and Research Hospital, Clinic of Family Medicine, Istanbul, Turkey

**Keywords:** COVID-19, vitamin B12 deficiency, homocysteine, COVID-19, vitamin B12, homocistein

## Abstract

**Background:**

COVID-19 disease affects the respiratory and cardiovascular systems. Vitamin B12 has been associated with A1AT, one of the protective factors of lung tissue, and homocysteine among the cardiovascular risk factors. Therefore we suggest that low vitamin B12 levels are associated with a disposition to COVID-19 infection. This study aims to determine whether there is a relationship between COVID-19 infection and serum vitamin B12 levels.

**Methods:**

This research is a case-control study. Seventy-six people with COVID-19 constituted the case group. Seventy-six people without COVID-19 formed the control group. Vitamin B12 and homocysteine levels of 152 patients included in the study were analyzed.

**Results:**

The odds ratio for vitamin B12 was 0.99 (0.978-0.995). When the value of the vitamin B12 variable decreases by one unit, the risk of COVID-19 increases by 1%. The odds ratio for homocysteine was 1.81 (1.414-2.325). When the value of the homocysteine variable increases by one unit, the risk of COVID-19 increases by 1.81 times. According to ROC analysis, when serum vitamin B12 is below 222.5 ng/L and homocysteine is above 13.7 mmol/L, it may increase the risk of COVID-19.

**Conclusions:**

We suggest that patients with low vitamin B12 levels and high homocysteine levels are more severely affected by COVID-19 infection.

## Abstract (sr)

**Uvod:**

Bolest COVID-19 utiče na respiratorni i kardiovaskularni sistem. Vitamin B12 je povezan sa A1AT, jednim od zaštitnih faktora plućnog tkiva, i homocisteinom među kardiovaskularnim faktorima rizika. Stoga predlažemo da je nizak nivo vitamina B12 povezan sa sklonošću ka infekciji COVID-19. Ova studija ima za cilj da utvrdi da li postoji veza između infekcije COVID-19 i nivoa vitamina B12 u serumu.

**Metode:**

Ovo istraživanje je studija slučaj-kontrola. Sedamdeset šest osoba sa COVID-19 činilo je grupu slučajeva. Kontrolnu grupu činilo je 76 osoba bez COVID-19. Analizirani su nivoi vitamina B12 i homocisteina kod 152 pacijenta uključena u studiju.

**Rezultati:**

Odnos izgleda za vitamin B12 bio je 0,99 (0,978-0,995). Kada se vrednost varijable vitamina B12 smanji za jednu jedinicu, rizik od COVID-19 se povećava za 1%. Odnos izgleda za homocistein bio je 1,81 (1,414-2,325). Kada se vrednost varijable homocisteina poveća za jednu jedinicu, rizik od COVID-19 se povećava za 1,81 puta. Prema ROC analizi, kada je serumski vitamin B12 ispod 222,5 ng/L, a homocistein iznad 13,7 mmol/L, to može povećati rizik od COVID-19.

**Zaključak:**

Predlažemo da su pacijenti sa niskim nivoom vitamina B12 i visokim nivoom homocisteina teže pogođeni infekcijom COVID-19.

## Introduction

Epidemiological studies show that cobalamin deficiency rates vary between 5% and 60% [1]. It has been stated that this change shows a positive correlation with age [2]. Vitamin B12 deficiency increases the risk of developing clinical pictures with high mortality, such as myocardial infarction and cardiac shock [3]. Vitamin B12 acts as a cofactor of the methionine synthase enzyme that provides the regeneration of methionine from homocysteine [4]. Methionine is one of the important amino acids involved in protein synthesis and also plays a role in methylation reactions by its S-adenosyl methionine (SAM) metabolite [5] [6]. In addition, it is one of the most potent antioxidants [7]. On the other hand, homocysteine causes ischemia by causing endothelial dysfunction and vasospasm mediated by oxidative stress [8] [9]. In addition, high levels of homocysteine have been associated with thromboembolic diseases [10].

COVID-19 disease is a respiratory infection involving the cardiovascular system, as well as the lungs [11] [12] [13]. One of the protective factors of lung tissue is alpha-1-antitrypsin (A1AT), a proteinase inhibitor [14]. In the literature, it was claimed in some studies that vitamin B12 treatment leads to an increase in A1AT levels [15]. In addition, vitamin B12 deficiency has also been associated with increased levels of homocysteine which is among the cardiovascular risk factors [16] [17] [18].

In light of this information, we think that low vitamin B12 levels are associated with a disposition to COVID-19 infection. Therefore, we wanted to determine whether there is a relationship between COVID- 19 infection and serum vitamin B12 levels.

## Materials and methods

### Study design and population selection

This research was designed as a case-control study. The sample size was determined for logistic regression analysis, considering the number of four independent variables, such as vitamin B12, age, gender, and body mass index. For each group, using the power analysis method, the minimum sample size of at least 38 which is needed to detect a significant difference when taken into account at 0.05 type-I error (Alpha), 0.35 effect size, 0,80 power (1-beta).

In this study, 152 patients admitted to the Istanbul Training and Research Hospital Family Medicine outpatient clinic for COVID-19 suspicion or postcovid follow-up between June and November 2020 were included and whose vitamin B12 levels were measured at the time of admission. The data were collected by scanning the hospital information system and patient records.

We determined that 395 patients had been admitted with suspicion of COVID-19 or for follow-up within two weeks after discharge during the six months examined in the study, and 208 patients’ serum vitamin B12 or homocysteine levels were not measured. A program named E-nabız records of 26 patients were not available. Another nine patients with comorbidities that may cause pseudo-elevation of vitamin B12 were excluded from the study. Consequently, 76 patients with positive polymerase chain reaction (PCR) tests or thoracic tomographic findings were included in the case group, and 76 patients with negative findings were included in the control group. Vitamin B12 and homocysteine levels of 152 patients included in the study were analyzed.

### Exclusion criteria

The patients who were diagnosed with COVID- 19 depending on a positive PCR test and/or thoracic tomography findings but vitamin B12 measurements were not performed, those with accompanying diseases that cause increased or decreased vitamin B12 levels or pseudo-elevation of vitamin B12 levels, and those who received vitamin B12 treatment were excluded from the study [19].

### Statistical analysis

The statistical analyses were performed using IBM SPSS Statistics software (version 25). Categorical data are expressed as numbers and percentages. The Chi-square test was used for the comparison of categorical groups. Numerical data were expressed as mean ± standard deviation (SD). The distribution of demographic data was analyzed by frequency tests, comparison of categorical data by chi-square test, and comparison of numerical data by independent sample t-test. The stepwise enter model was used in the binary logistic regression test to evaluate the effect of vitamin B12 and homocysteine on COVID- 19. The cut-off values were calculated by ROC analysis for vitamin B12 and homocysteine. Skewness and kurtosis analyses were used to conform the data to the normal distribution A value of p < 0.05 was considered statistically significant. This study was approved by the local Clinical Research Ethics Committee (Numbered decision: 2593).

## Results

There were no significant differences between the groups regarding body mass index (BMI) and comorbidities. However, we found that the means of age and the rate of males were statistically significantly higher in the case group compared to the control group (Table 1). In the case group, serum vitamin B12 levels were found to be statistically significantly lower than in the control group (p < 0.001) (Table 1). In the control group, serum homocysteine levels were found statistically significantly lower than in the case group (p < 0.001) (Table 1).

**Table 1:** Comparison of the gender, comorbidity, age, BMI, vitamin B12 and homocysteine between case and control groups. SD: Standard Deviation; BMI: Body Mass Index; *The statistical significant different was accepted as p <0.05

Characteristics of participants		Case (n=76)	Control (n=76)	p
Gender (n;%)	Female	32; 33.3	64; 66.7	< 0.001*
	Male	44; 78.6	12; 21.4	
Comorbidity (n;%)	Yes	38; 55.9	30; 44.1	0.253
	No	38; 45.2	46: 54.8	
Age (year) (Mean ± SD)		55.55 ± 14.12	49.78 ± 11.26	< 0.001*
BMI (Mean ± SD)		26.78 ± 3.22	25.87 ± 3.81	0.112
Vitamin B12 (Mean ± SD) (ng/L)		190.66 ± 90.19	279.74 ± 100.09	< 0.001*
Homocysteine (Mean ± SD) (mmol/L)		18.21 ± 5.41	11.15 ± 2.36	< 0.001*

Binary logistic regression analysis was performed by considering the different parameters between the case and control groups. The age, gender, and homocysteine variables, which were different between the case and control groups, were included in the analysis. The model is significant in the analysis in which all the independent variables were added because the Cox-Snell R2 and Nagelkerke R2 values are bigger than the 0.2 level. Except for the step in which vitamin B12 was added, the model’s goodness of fit is acceptable since the p-values are bigger than 0.05 in the Hosmer-Lemeshow test. Changes in -2 Log Likelihood values (Chi-square, p < 0.05) are significant in vitamin B12, homocysteine, and gender steps. These results show that the logistic regression analysis is generally valid. In the presence of independent variables, the predictive percentage of the logistic regression model is 88.2%, which is quite high (Table 2).

**Table 2:** Distribution of data showing the validity of the logistic regression analysis. *The statistically significant difference was accepted as p < 0.05

Metod=Enter: <br>stepwise	-2 Log <br>Likelihood	Omnibus Tests of <br>Model Coefficients		Cox and Snell <br>R Square	Nagelkerke <br>R Square	Hosmer and <br>Lemeshow <br>Test	Predicted <br>percentage
		Chi-square	p				
Beginning	210.717						50.0
+Vitamin B12	178.946	31.771	< 0.001*	0.189	0.251	< 0.001*	72.4
+Homocysteine	106.828	72.118	< 0.001*	0.495	0.660	0.692	84.2
+Age	106.558	0.270	0.604	0.496	0.661	0.638	86.2
+Gender	88.410	18.148	< 0.001*	0.553	0.737	0.834	88.2

**Figure 1:**
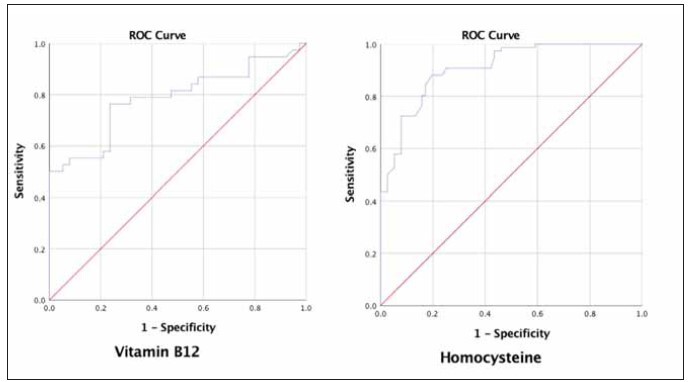
ROC curve was applied to evaluate cut-off values for vitamin B12 and homocysteine in patients with positive and negative COVID-19.

The odds ratio for vitamin B12 was 0.99 (0.978–0.995). When the value of the vitamin B12 variable decreases by one unit, the risk of COVID-19 increases by 1% (Table 3). The odds ratio for homocysteine was 1.81 (1.414–2.325). When the value of the homocysteine variable increases by one unit, the risk of COVID-19 increases by 1.81 times (Table 3). The odds ratio for gender was 19.98 (3.979– 100.356). When the reference group of women are taken, the risk of COVID-19 increases by 19.98 times in men (Table 3). According to ROC analysis, when serum vitamin B12 is below 222.5 ng/L and homocysteine is above 13.7 μmol/L, it may increase the risk of COVID-19 (Table 4 and Figure 1).

**Table 3:** Logistic regression analysis showing the relationship between COVID-19 and variables of vitamin B12, homocysteine, age, and gender. ^a^Dependent Variable: COVID-19; B: Estimated Coefficients; CI: Confidence Interval; SE: Standard Error; *Logistic regression analysis is significant at the p < 0.05 level (2-tailed).

COVID-19a	B	SE	Wald	p	Risk (Odds) <br>coefficient (Exp B)	95% CI for (Exp B)	
						Lower	Upper
Vitamin B12	-0.013	0.004	9.941	0.002*	0.99	0.978	0.995
Homocysteine	0.595	0.127	22.025	< 0.001*	1.81	1.414	2.325
Age	-0.039	0.024	2.761	0.097	0.96	0.918	1.007
Gender (1: Women/Men)	2.995	0.823	13.228	< 0.001*	19.98	3.979	100.356

**Table 4:** ROC analysis and cut-off value for vitamin B12 and homocystein. CI: Confidence Interval; *ROC analysis is significant at the p < 0.05 level (2-tailed).

	Area Under <br>the Curve	95% CI		p	Sensitivity	Specificity	cut-off<br>value
		Lower	Upper				
Vitamin B12	0.785	0.711	0.860	< 0.001*	0.763	0.763	222.5
Homocysteine	0.909	0.864	0.953	< 0.001*	0.882	0.803	13.70

## Discussion

Similar to the literature, we found that the means of age and the rate of males were higher in the case group compared to the control group. This finding is noteworthy in supporting the results indicating that men and the elderly are more affected by the COVID-19 disease and that our study fits the normal population [20] [21].

In our study, when the value of the vitamin B12 variable decreases, the risk of COVID-19 increases. Although non-explained the mechanism of the relationship between vitamin B12 and COVID-19 fully, there are some studies that emphasized vitamin B12 supplementation reduces the symptoms caused by COVID-19 in the literature [22] [23]. In addition, when serum vitamin B12 is below 222.5 ng/L it may increase the risk of COVID-19. In the literature, it has been suggested that vitamin B12 treatment increases A1AT levels [15]. A1AT protects the lung parenchyma and defense against infectious agents [14]. In addition, a study in Italy suggested that the distribution of the COVID-19 pandemic and A1AT insufficiency coincided geographically. In this case, it could not be explained by a random relationship [24]. In light of this information, low vitamin B12 levels decrease A1AT synthesis and increase susceptibility to COVID-19 infection.

Methyl malonyl coenzyme A is transformed into succinyl coenzyme A by methyl malonyl coenzyme A mutase enzyme. Succinyl coenzyme A, which mediates fatty acid beta-oxidation and glucose production through gluconeogenesis, cannot be formed in vitamin B12 deficiency [25]. This failure deprives the organism of critical energy pathways and cannot produce the energy it needs. As a result, it could be thought that the long-term fatigue and weakness seen in patients in the postcovid period may be associated with low vitamin B12 levels.

In our study, when the value of the homocysteine increases, the risk of COVID-19 increases. In addition, when serum homocysteine is above 13.7 μmol/L, it may increase the risk of COVID-19. In the literature, it has been shown that factor V, factor VIIa, and factor XII activities increased, protein C and antithrombin inhibited, tissue factor release stimulated, and thrombomodulin and heparin sulfate release decreased when the level of homocysteine increased [26]. In addition, high homocysteine levels increase the susceptibility to coagulation and ischemia [8] [9] [10]. This information suggests that cardiovascular events and myocardial ischemia in COVID-19 patients may be related to high homocysteine levels [16] [17] [18]. In addition, we think that elevated homocysteine levels increase the tendency to coagulation and contribute to the long covid process seen in some patients.

## Conclusion

In light of this information, we suggest that increased vitamin B12 levels play an active role in protecting against COVID-19 infection by reducing homocysteine levels, contributing to energy production, and maybe increasing A1AT levels. We suggest that COVID-19 severely affects those with low vitamin B12 levels and high homocysteine levels. Further studies with larger samples are needed.

## Dodatak

### List of abbreviations

A1AT: Alpha-1 antitrypsin;<br>BMI: Body mass index;<br>PCR: polymerase chain reaction;<br>SAM: S-adenosyl methionine

### Funding

The authors declare no funding.

### Conflict of interest statement

All the authors declare that they have no conflict of interest in this work.
